# Plant movements: navigating the light environment

**DOI:** 10.1093/plphys/kiag110

**Published:** 2026-03-04

**Authors:** Sanne E A Matton, Lisa Oskam, Ronald Pierik

**Affiliations:** Laboratory of Molecular Biology, Wageningen University and Research, Wageningen, the Netherlands; Laboratory of Molecular Biology, Wageningen University and Research, Wageningen, the Netherlands; Laboratory of Molecular Biology, Wageningen University and Research, Wageningen, the Netherlands

## Abstract

Plants use light both as a resource for photosynthesis and as a signal about their environment. In response to light cues, plants can move their organs via directional growth driven by cell expansion. In dense vegetation where light is available in spatially heterogeneous patterns, plants need to navigate this space to improve the position of their photosynthetic tissues. In canopies blue light irradiance and red to far-red light ratio decrease due to absorption by chloroplasts, and these changes regulate distinct processes within the plant. Changes in light environment are detected by cryptochrome and phytochrome photoreceptors, both regulating phytochrome interacting factors (PIFs) and thereby enhancing elongation in hypocotyls, stems, and leaves and inducing upward leaf movement (hyponasty). An additional class of photoreceptors, phototropins, decodes horizontal light gradients to produce directional growth toward the light source (phototropism). Here we review the current state of knowledge on these differential growth responses to light cues, with specific emphasis on the regulatory pathways that translate light signaling into differential cell expansion. Downstream of the photoreceptors, the phytohormone auxin induces cell growth in shoot tissues, but also other phytohormones contribute to balancing light responses. Cell expansion is regulated primarily at the level of cell walls, and a comparison of different transcriptome datasets reveals that only a small group of cell wall–modifying genes are tightly regulated by shade cues. It remains poorly understood which cell layers are causal to the initiation of cellular expansion. Here we combine insights from different differential growth behaviors in different species and organs to generate different hypotheses for the cellular underpinnings of light-driven leaf movements.

## Introduction

Everyone has read the sentence “plants are sessile organisms” perhaps one too many times. Although indeed rooted at their spot, plants in fact move their organs a lot, albeit at a speed that our eyes often dismiss. Throughout the life course of a plant, movement is required to ensure survival and reproduction. A just-germinated seedling has its hypocotyl tip bent downward in a so-called apical hook to protect the cotyledons and meristem while pushing through soil. When it reaches the light, the apical hook opens, and the cotyledons that move upward through this process subsequently unfold. Organ movement will continue throughout the following life stages in a search for light while navigating the canopy, all the way into the reproductive stage where flowers open and close. However, movement of plants is limited by the rigidity of the cell wall. Therefore, plants have to either employ differential growth within still-growing tissues (Jonsson et al. 2023) or depend on specialized structures that have the potential to rapidly expand and contract (Ueda et al. 2019). Pulvini in, for example, legumes are an example of the latter reversible category, potentially evolved to protect (indirectly) against herbivory (Minorsky 2019; Feng et al. 2023). Venus flytraps are another famous example of rapid reversible contraction (Forterre et al. 2005) but display a much slower release of the trap (Zeng et al. 2024), highlighting that the speed of expansion and contraction can be asynchronous. Both examples allow movement without the need for actual permanent organ growth due to the specialized structures present that expand and contract.

However, several movements involve differential growth, which constitutes a 1-sided change in growth rate in an organ. This could be either via a reduction in growth on the inner corner of the bend or an increase in growth on the outer side. Most described instances of differential growth in plants are based on enhanced unidirectional expansion (cell elongation) rather than additional cell divisions or of the inhibition of elongation on the opposite side (Gendreau et al. 1997; Polko et al. 2012; Su et al. 2017; Legris and Boccaccini 2020). When the organ grows, elongating cells retain their additional length compared with the opposite side. The implication of this is that this type of movement is confined to growing tissue and will dampen when the growth potential has reached its maximum. It also implies that there can be generic organ length phenotypes associated with diurnal movements, such as for leaf angles (Woodley of Menie et al. 2019; Oskam et al. 2024) and heliotropism (Atamian et al. 2016). On the contrary, various flowering species open and close their petals repeatedly over the course of several days (Van Doorn and Kamdee 2014). For example, the abaxial and adaxial epidermal layers of petals of waterlilies are described to elongate and shrink during the day (Ke et al. 2018), thus enabling the repeated opening and closing of the flowers without the need for prolonged total organ expansion.

Opening and closing of flower petals is partially controlled by photo period and light quality, as are diurnal leaf movements (Woodley of Menie et al. 2019; Liu et al. 2022). Shade is a broader term used to describe canopy light conditions with a particularly strong decrease in blue and red-light irradiance combined with a relative enrichment of reflected and transmitted far-red light. Here, we review how plants use shade cues to navigate the canopy and how these movements are dependent on the site and direction of light signal perception. For growing organs, such as internodes or leaves, elongation involves cell expansion and cell proliferation, with the balance between the two depending on their developmental stage. Bending responses toward light that typically occur over time scales of hours are associated with differential cell expansion between two sides of an organ.

Here, we will set the stage with a description of the light dynamics in plant vegetation, their perception through photoreceptors, and the resulting shade avoidance responses. This will be followed by a review of cell expansion in the context of shade-induced hypocotyl elongation. We then build on this foundation by exploring two light-driven bending responses further in depth: phototropism and hyponasty, that are based on differential growth, and secure light interception in crowded vegetation.

## Light and canopy dynamics

From germination to reproduction, plants depend on light not just for photosynthesis but also as an information layer. Photosynthesis is powered by all wavelengths in the visible light, but especially well by red and blue light, the two colors that are preferentially absorbed by chlorophyll (Liu and van Iersel 2021). Light that is reflected or transmitted by leaves is relatively rich in green and in far-red light. From top to bottom in the canopy the total light irradiance decreases, and the spectral composition changes in favor of far-red and green light. In addition to the photosynthetic pigments, plants express photoreceptors to monitor light quality, irradiance, duration, and direction.

Plant vegetations, be it grasslands or forests, natural or agricultural, are characterized by light conditions that are highly dynamic in space and time. The vegetation develops vertically by plant growth, causing an ever-changing light dynamic (Huber et al. 2021). Solar position, clouds, and wind subsequently lead to more or less predictable, changes in light distribution through the canopy (Smith and Berry 2013; Morales and Kaiser 2020; Sellaro et al. 2024). In both sinusoidal variations in irradiance upon dawn and dusk and fluctuations mimicking clouds, a clear difference in photosynthetic responses is found compared with constant light conditions (Alameldin and Montgomery 2023). Generally speaking, the lower in the vegetation, the lower the light intensities and the more shade-acclimated the leaves typically are. Nevertheless, canopy heterogeneity can lead to temporal spikes considering very high irradiance, sunflecks, potentially damaging the photosynthetic machinery of exposed leaves (Pospíšil 2016). Chloroplasts typically move away from such temporary high light exposure toward the walls parallel to the blue light direction (Wada 2013; Łabuz et al. 2022). The blue light gradients that occur in leaves also regulate leaf morphological adjustments, such as flattening of leaves that can help improve light interception (Legris et al. 2021). The heterogeneity of light composition and irradiance in space and time affects plant photosynthesis. To ensure favorable light exposure, plants use information from their photoreceptors to navigate the light environment through (differential) growth plasticity. This repositioning of shoot organs helps to move organs away from shade and expose more leaf area to light, thus improving photosynthesis by increasing light captured per unit area.

## Photoreceptor signaling

Photoreceptors are tightly connected to downstream signaling networks affecting a wide range of processes within the plant. Activity of photoreceptors is determined by absorption of specific wavelengths. Blue light (400 to 500 nm) is sensed primarily by cryptochromes and phototropins, red (R, 600 to 700 nm) and far-red light (FR, 700 to 780 nm) by phytochromes (Huq et al. 2024), and UV-B (280 to 315 nm) by UV Resistance Locus 8 (UVR8) (Rizzini et al. 2011) and phototropins (Vandenbussche et al. 2014). Active phytochromes repress phytochrome interacting factors (PIFs) (Casal 2013), a subgroup of bHLH transcription factors (Leivar and Monte 2014), through direct interaction. Active cryptochromes can also affect PIF function by direct interaction (Pedmale et al. 2016). PIFs regulate expression of a wide variety of target genes, including genes involved in production, signaling, and transport of the phytohormone auxin, an essential factor in plant growth and development (Han et al. 2023; Vanneste et al. 2025). Furthermore, signaling, biosynthesis, and/or transport of many other hormones, notably gibberellins (Henschel et al. 2021), brassinosteroids (Kozuka et al. 2010), abscisic acid (Michaud et al. 2023), jasmonic acid, salicylic acid, and strigolactones, are also regulated by light sensing (Fernández-Milmanda and Ballaré 2021; Gautrat et al. 2025). Of these hormones, brassinosteroids, auxin, and gibberellin cooperatively regulate gene expression through a proposed BAP/D transcription factor module (Oh et al. 2014; Chaiwanon et al. 2016). The consequential degree of cellular response depends on the relative activity of these different hormones and their target transcription factors. Since hormones are mobile, their fundamental roles in processing photoreceptor information enables transmission of light information over spatial scales. This is also true for the mobile transcription factor HY5 (Oyama et al. 1997; Toledo-Ortiz et al. 2014) that can translocate from shoot to root (Chen et al. 2016) and is strongly light-responsive through its interaction with the light-sensitive E3 ubiquitin ligase COP1 (Ponnu et al. 2019; Yadav et al. 2020; Mao et al. 2021). To summarize, plants can detect and physiologically respond to different wavelengths of light with high precision and do so via several different photoreceptors, proteins, and phytohormones.

## Shade avoidance responses to canopy signals

**Figure 1:**
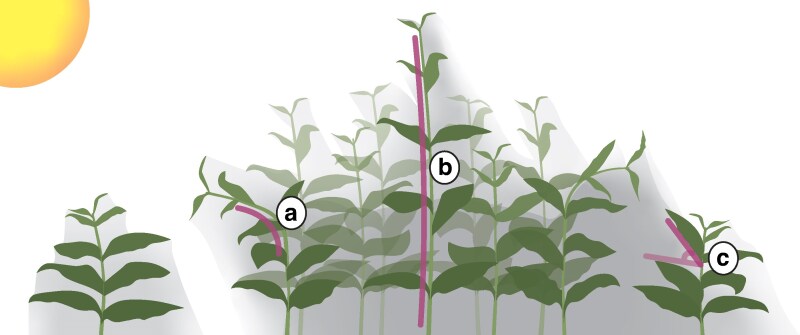
Shade responses in plants growing in a canopy. A single non-shade–exposed plant on the left side highlighting general phenotypic development, and a canopy on the right with plants displaying 3 shade avoidance traits (purple lines). Gray shading indicates a reduction in irradiance. **a)** Phototropic bending of the stem toward higher irradiance. **b)** Increased internode elongation, growing toward higher irradiance. **c)** Hyponasty, upward differential growth of leaves toward higher irradiance.

Many species need light to germinate and, once germinated, use it to develop chloroplasts and become photoautotrophic (Gommers and Monte 2018). The cotyledons are carried by the embryonic stem, the hypocotyl, that can bend toward the light, a phenomenon known as phototropism. Although less well studied, plants in later life stages can also be phototropic (Matsuzaki et al. 2006; Kagawa et al. 2009; López Pereira et al. 2017) (Fig. 1a). Phototropism is primarily a response to horizontal blue light gradients that are detected by phototropins (Liscum et al. 2014; Fankhauser and Christie 2015; Legris and Boccaccini 2020) but can also involve red and far-red light (Ballaré et al. 1990; Goyal et al. 2016) under more realistic canopy light scenarios. A reduction in the red and far-red light ratio (R:FR) or blue light, such as occurring in vegetation shade, leads to an increase in elongation growth in light-grown hypocotyls (Cole et al. 2011; Li et al. 2012; Procko et al. 2014, 2016; Pedmale et al. 2016; Paulišić et al. 2021), petioles (Djakovic-Petrovic et al. 2007; Keller et al. 2011; de Wit et al. 2015), and internodes (Wu et al. 2019; Courbier et al. 2021; Lyu et al. 2021) in various species (Fig. 1b). In petioles, the shade signal-induced increase in growth takes place over the entire radial axis. On top of this, the abaxial side elongates more than the adaxial in shade, and this differential growth leads to upward bending of the petiole, a process called hyponasty that elevates the leaves toward the light from above (Michaud et al. 2017; Pantazopoulou et al. 2017) (Fig. 1c). In addition to this vertical movement, petioles can also bend sideways (Michaud et al. 2017), potentially enabling plants to escape local shade patches. While petiole elongation and hyponasty co-occur, hyponasty is fully dependent on low R:FR signaling in the leaf tip, whereas petiole elongation is not (Pantazopoulou et al. 2017). The specific light signals, plant tissues, and growth stages of the plant determine which members of the PIF family transduce the information into growth plasticity (Pfeiffer et al. 2014). PIF4 and PIF5 have important roles in low R:FR-induced petiole elongation (Pantazopoulou et al. 2017) and blue light-mediated petiole and hypocotyl regulation (Keller et al. 2011; Pedmale et al. 2016). PIF7 is especially important for low R:FR-induced auxin synthesis (Li et al. 2012) in the cotyledons or lamina, which can subsequently be translocated to responding tissues (Keuskamp et al. 2010; Procko et al. 2014; Kohnen et al. 2016; Michaud et al. 2017; Pantazopoulou et al. 2017; Küpers et al. 2023).

Hyponastic growth, vertical stem and hypocotyl elongation, reduced branching to favor main shoot elongation (apical dominance), and accelerated flowering are all categorized under the shade avoidance syndrome (Fernández-Milmanda and Ballaré 2021; Casal and Fankhauser 2023; Gautrat et al. 2025). Although often treated as a separate phenomenon, phototropism is in fact an integral part of the shade avoidance syndrome when considering real life vegetations.

Shade avoidance can enhance plant fitness in heterogeneous, natural vegetations but can also lead to losses in crop yield if it involves a divergence of plant investments away from harvestable organs (Keuskamp et al. 2010; Crepy and Casal 2015; López Pereira et al. 2017; Bongers et al. 2018; Pantazopoulou et al. 2021). Responses to shade cues can differ greatly between closely related species (Gommers et al. 2017; Molina-Contreras et al. 2019) and even between cultivars (Liu et al. 2016; Mughal et al. 2025) and are sensitive to other environmental parameters. Sunflecks of UV-B light dampen low R:FR-induced hypocotyl elongation via regulation of phytochromes, cryptochromes, and UVR8 (Belmonte et al. 2025). Moreover, nitrogen availability in the soil via cytokinin signaling (van Gelderen et al. 2021; Gautrat et al. 2024) and salt stress via abscisic acid and brassinosteroids (Hayes et al. 2019) modulate low R:FR-induced hypocotyl elongation. Interestingly, although far-red light is mostly seen as a signal only, it can also directly promote biomass growth in some species through absorption for photosynthesis (Zhen and Bugbee 2020a, 2020b; Zhen et al. 2022; Huber et al. 2024; Jans et al. 2025). It is important to note that FR-related promotion of biomass is limited to enhancement of photosynthesis, as FR light by itself is not enough to elicit activation of the photosystems. This relatively recent insight suggests that photosynthesis in different layers of vertically structured vegetation is not just determined by the extinction of visible light from top to bottom but also by the relative enrichment of far-red light. Together, canopy signals shape plant growth and movement throughout their lifecycle.

## Light-induced cell expansion

Movement as a response to light is driven by cell expansion (Jonsson et al. 2023; Krahmer and Fankhauser 2024), with a dominant role in hypocotyls for epidermal cells in facilitating and restricting growth (Savaldi-Goldstein et al. 2007; Procko et al. 2016; Kelly-Bellow et al. 2023). Cell expansion is the result of primarily two processes: turgor pressure and loosening of primary cell walls. Turgor pressure is created by water uptake and storage in vacuoles, which can take up most of the cell volume (Cosgrove 2024). Pressure is built up by the constraint on cellular volume increase caused by the rigid cell wall that encapsulates the plasma membrane (Coen and Cosgrove 2023). The core constituents of primary cell walls are cellulose, hemicellulose, and pectins, all polysaccharides (Ali and Traas 2016). Primary cell wall cellulose is structured into cellulose microfibrils embedded in a matrix of hemicellulose and pectins. Both the matrix and the cellulose microfibrils can form fibrous networks that carry severe loads to resist turgor pressure (Cosgrove 2024). The turgor pressure creates a tensile stress within the primary wall and, depending on the strength of different zones of the wall, this can lead to deformations or growth in a directional manner.

**Figure 2:**
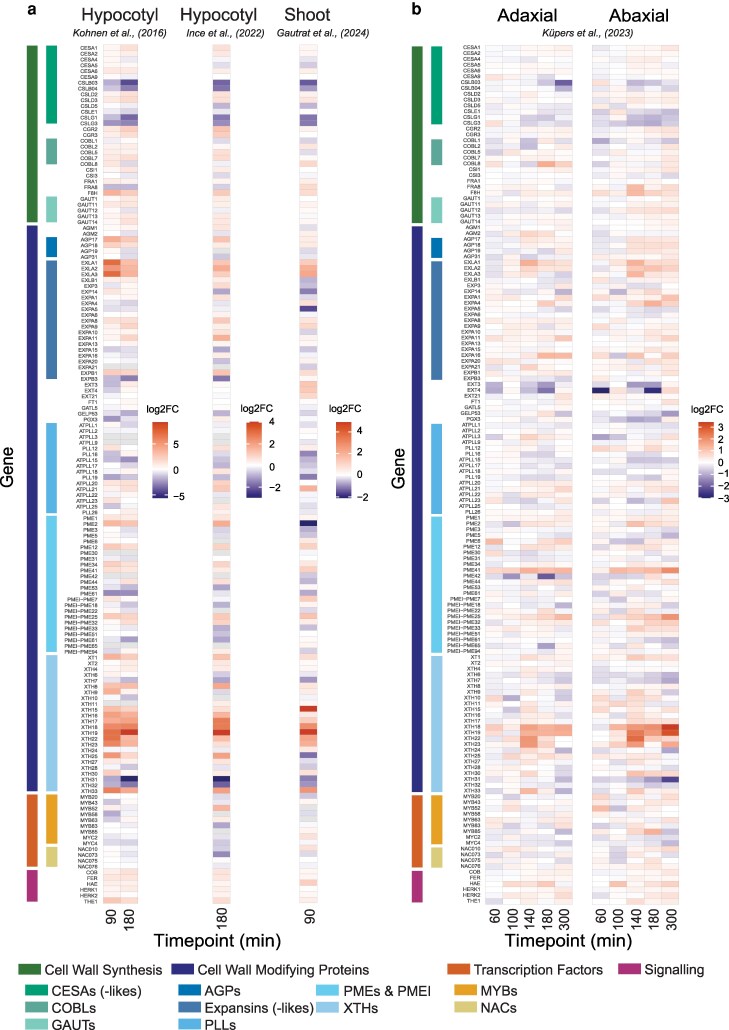
Robust changes in expression of genes related to cell wall changes upon supplemental FR exposure. **a)** Three independent experiments on seedlings, harvested around a similar time frame. **b)** Petioles of adult plants separated in abaxial or adaxial sides, harvested at several timepoints. Harvested tissue and original study are indicated above, timepoint of harvesting after the start of supplemental FR treatment is indicated below. Color codes on the left indicate different categories of genes related to cell wall adjustments, with subcategories of genes with 4 or more members.

The flexibility of cell walls and zones within the cell wall can be modulated by cell wall modifying proteins (CWMPs) (Peaucelle et al. 2008, 2011, 2015; Harada et al. 2011; Leroux et al. 2015). CWMPs act on specific elements, such as pectins, in the cell wall and temporarily loosen the cell wall, allowing for cell volume increase driven by turgor pressure (Sasidharan et al. 2011; Cosgrove and Jarvis 2012). Examples of CWMPs include expansins, xyloglucan-transglucosylase/hydrolases (XTHs), and pectin methylesterases (PMEs). Regulation of CWMPs occurs in response to a variety of environmental inputs (Sasidharan et al. 2011). Light signaling regulates the expression and physiological activity of several CWMPs (Pelletier et al. 2010; Sasidharan et al. 2010; Pfeiffer et al. 2014; Sénéchal et al. 2024). Transcriptome studies on shade signal-induced hypocotyl elongation show gene ontology (GO) enrichment of cell wall modification and organization, cell wall biogenesis, and cell growth (Kohnen et al. 2016; Pedmale et al. 2016; Ince et al. 2022; Sénéchal et al. 2024) with both overlapping and unique expression patterns in low blue vs low R:FR treatments (Ince et al. 2022). We plotted the supplemental FR-induced expression changes of genes encoding CWMPs, cell wall synthesis, or cell wall signaling from 3 different RNA sequencing datasets on Arabidopsis seedling hypocotyl elongation (Kohnen et al. 2016; Ince et al. 2022; Gautrat et al. 2024) in Fig. 2a. Interestingly, only a few specific members within those gene families are differentially regulated in FR enriched light, and the expression patterns are strongly conserved across multiple independent experiments (Kohnen et al. 2016; Ince et al. 2022; Gautrat et al. 2024) (Fig. 2a). It is also clear that within the short time-frame of these experiments, cell wall synthesis genes are not activated. Whether cell wall synthesis genes would be activated at a later timepoint is currently unknown. PIFs, cryptochromes, and HY5 have been shown to regulate expression of cell wall modifying genes (de Wit et al. 2015; Pedmale et al. 2016; Mao et al. 2021). It was also found that mutants in pectin and xyloglucan biosynthesis are impaired in cell expansion during low R:FR-induced hypocotyl elongation (Sénéchal et al. 2024). In leaves, low R:FR, as well as the combination of low R:FR and low blue and total irradiance, induce several *XTH* genes, with different genes being regulated dependent on the leaf tissue (Sasidharan et al. 2014; de Wit et al. 2015) and at least XTH15 and XTH17 being functionally required for expression of shade avoidance in Arabidopsis (Sasidharan et al. 2010).

Low R:FR initiates YUCCA-dependent de novo synthesis of auxin in cotyledons and leaves (Procko et al. 2014; Michaud et al. 2017; Pantazopoulou et al. 2017) that is translocated to the hypocotyl or petiole where differential growth can be induced. This auxin transport is dependent on changes in (polar) localization and abundance of auxin export proteins PIN-FORMED 3 (PIN3), PIN4, and PIN7 (Keuskamp et al. 2010; de Wit et al. 2015; Kohnen et al. 2016; Pedmale et al. 2016; Michaud et al. 2017; Pantazopoulou et al. 2017; Boccaccini et al. 2020). Via members of the family of small auxin upregulated RNAs (SAURs), auxin regulates phosphorylation of the plasma membrane P- type H^+^-ATPase (Fendrych et al. 2016; Stortenbeker and Bemer 2019; Lin et al. 2021), thereby lowering the pH of the cell walls. This acidification helps activate CWMPs such as expansins (Cosgrove 2015) that can temporarily modify the cell wall, thus allowing cell elongation.

## Growing toward blue light: phototropism

Seedling phototropism toward unidirectional blue light is a well-studied response to a blue light gradient through the hypocotyl, often studied in etiolated (ie dark-grown) seedlings (Liscum et al. 2014; Fankhauser and Christie 2015; Legris and Boccaccini 2020; Haga and Sakai 2023). Bending toward unidirectional blue light requires differential cell elongation between the light-exposed and non-exposed side of the hypocotyl itself. Seedlings can also display bending toward UV-B light in a UVR8-dependent manner (Vandenbussche et al. 2014). The two phototropins present in Arabidopsis, phot1 and phot2, change activity at distinct light intensities, enabling plants to respond to blue light gradients at both low and high light intensities (Haga and Sakai 2023) at both seedling and adult stages (Kagawa et al. 2009). The gradient starts from directional blue light that is subsequently scattered between cells and internal air spaces and partially absorbed in the hypocotyl, leading to an internal gradient inside the organ (Nawkar et al. 2023). In response to the light gradient, there is differential activation of phototropin photoreceptors between the shaded and illuminated sides of the hypocotyl, thus changing the phot1-NPH3 complex, consisting of the phot1 photoreceptor and the signaling protein ROOT PHOTOTROPISM2, NONPHOTOTROPIC HYPOCOTYL3 (NPH3) (Haga and Sakai 2023; Sakai et al. 2025; Zhu et al. 2025). This differential phototropin activation sets up an auxin gradient that is the inverse of the light gradient between the illuminated and non-illuminated side (Goyal et al. 2013) via PINOID protein kinase–dependent polarization of the auxin transporter proteins (Benjamins et al. 2001), such as PIN3 (Ding et al. 2011). Auxin rapidly initiates phosphorylation of plasma-membrane H^+^-ATPases (Lin et al. 2021), such as AHA1 and AHA2, whose activity is maintained by auxin-induced *SAURs*, for example, SAUR19, that repress the type 2C protein phosphatase PP2C-D (Spartz et al. 2014). The resulting apoplastic acidification subsequently promotes hypocotyl cell growth (Fendrych et al. 2016) but within limits; if the hypocotyl's apoplastic pH drops below approximately 4.4 in Arabidopsis hypocotyls, for example, by excessive auxin-induced activation of AHAs, it becomes growth limiting (Wang et al. 2025). This is exactly why apoplastic over-acidification inhibits cell expansion is currently unknown.

Although phototropism is studied often in etiolated seedlings, the response is also apparent in green, fully deetiolated seedlings. In those cases, the phytochrome and cryptochrome photoreceptors suppress phototropic bending, but this can be alleviated by canopy shade cues such as low R:FR (Goyal et al. 2016) and reduced overall blue light irradiance (Boccaccini et al. 2020). The R:FR-mediated modulation of phototropic bending toward blue light acts via the phyB-PIF module described earlier, leading to enhanced auxin synthesis in the cotyledons (Goyal et al. 2016), likely adding to the transverse auxin gradient in the hypocotyl. The promotive effect of blue light depletion relies on PIFs as well and appears to also strengthen the transverse auxin gradient in the hypocotyl (Boccaccini et al. 2020). This effect occurs via CRY1 inactivation (Boccaccini et al. 2020) and CRY1 also interacts with TRANSPARENT TESTA GLABRA 1 to modulate phototropism under high intensity blue light (Wang et al. 2024). Phototropic bending of light-grown seedlings also integrates regulation from the evening complex of the circadian clock through control by ELF3 and LUX, upstream of PIFs (Cobb et al. 2025). Indeed, it was observed that phototropic potential is not constant over the day but peaks around the middle of the subjective day and decreases toward the subjective night, in circadian free-running experiments (Cobb et al. 2025). Within limits, the auxin gradient across the hypocotyl thus results in differential cell elongation between the two sides. Accordingly, hypocotyl phototropism is thought to occur primarily by differential auxin accumulation and response in the epidermal cells on opposite sides. Indeed, directional blue light increases the auxin signal reported by the synthetic DR5 reporter on the non-illuminated side (Ding et al. 2011). This enhanced auxin signaling thus promotes epidermal cell elongation and bending the hypocotyl away from the shaded side, toward the light source.

## Leaves navigating the canopy: hyponasty

Petioles moving upward require differential elongation of cells on opposing sides. Based on the light extinction model for phototropism, it is tempting to speculate that similar mechanisms of light gradients within a tissue leading to local auxin patterns could be involved in controlling organ bending in later stages of plant development. However, differential growth in the case of hyponasty does not reflect a local light gradient as with phototropism but rather a response driven by a remote light signal. Moreover, auxin gradients are often (Park et al. 2019; Küpers et al. 2023) but not always (Pantazopoulou et al. 2023) involved in vertical leaf movement. Upon low R:FR perception in adult leaves through phyB, PIF7 is activated and induces de novo auxin synthesis in the leaf tip via *YUCCA* activation (Pantazopoulou et al. 2017; Küpers et al. 2023). Auxin is subsequently translocated to the petiole base in a PIN-dependent manner (Michaud et al. 2017; Pantazopoulou et al. 2017), where it accumulates especially at the abaxial side (Küpers et al. 2023). The auxin concentration pattern formed between the abaxial and adaxial side is reflected in the different levels of upregulation between the abaxial and adaxial petiole of genes that encode cell wall modifying proteins, such as XTHs and expansins (Küpers et al. 2023) (Fig. 2b). Some of the CWMP genes may also be direct targets of PIF4 and 5 in the petiole (Hornitschek et al. 2012; Pedmale et al. 2016). Interestingly, only a few of the members of these large families are transcriptionally regulated, and members of other major CWMP gene families, such as PMEs, seem not regulated at all in response to FR enrichment (Fig. 2a and b). Also, genes associated with cell wall synthesis are hardly regulated in these tissues, despite the enhanced cell expansion that would require additional cell wall strengthening at some point (Fig. 2). These expression patterns show a high similarity to the expression patterns in hypocotyls when exposed to supplemental FR (Fig. 2a and b). This could indicate that much of the differential growth response to remote FR signaling is regulated at the cell expansion level, primarily through XTHs and expansins. It should, however, be noted that parts of cell wall regulatory circuit remain invisible in gene expression studies. For example, cell wall acidification through auxin-induced SAURs will likely activate CWMPs that are already in the apoplast, thus leading to cell wall loosening without additional CWMP gene expression. Additionally, the timing of regulation could be beyond the scope of the current gene expression studies.

**Figure 3:**
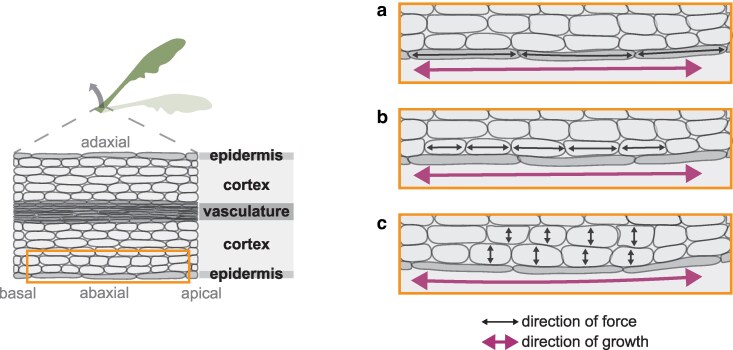
Three hypothetical scenarios of low R:FR-induced petiole hyponasty. Left: hyponastic leaf and longitudinal section of petiole base. Right: zoom in on the abaxial side of longitudinal petiole section for a, b, and c. **a)** Longitudinal cell elongation of epidermal cells drives growth. **b)** Longitudinal cell elongation of cortex cells bordering epidermal cells drives growth. **c)** Cortex cell expansion exerts a force on overlaying epidermal cells and thereby drives bending through curvature.

As with phototropism, differential epidermal cell elongation between the radially opposite sides of the Arabidopsis petiole, in this case the basal section of the abaxial and adaxial side, has been reported during hyponastic leaf movement (Polko et al. 2012; Küpers et al. 2023). It is, however, unknown if the epidermis is equally dominant in controlling these bending plasticities in adult organs as it is in seedling hypocotyls. In an Arabidopsis study on leaf hyponasty, cellular auxin distributions were quantified with the fluorescent R2D2 ratiometric reporter. It was observed that auxin accumulated on the abaxial compared with the adaxial side of the petiole through all tissue layers from the deeply lying bundle sheath to the epidermis (Küpers et al. 2023). In phototropic seedling hypocotyls, on the other hand, the auxin response reporter DR5 identified a rather specific enhancement of auxin response in the epidermis of the non-illuminated side and much less so in the inner tissues (Ding et al. 2011). Although in petiole hyponasty cell length changes of the epidermis were reported, they can still emerge from different underlying processes. For example, leafy head formation of Chinese cabbage follows from active differential growth of the inner parenchyma tissues that, by restriction by the upper and lower epidermis, forces a curvature of the leaf (Liu et al. 2024). Likewise, the opening of petals in rose flowers is driven by cell expansion of parenchyma cells between the adaxial epidermis and vascular bundles (Cheng et al. 2021). These examples indicate that even though the epidermis is a highly relevant tissue facilitating differential growth, the driving force could still be other, inner, tissues as much as the epidermis itself. Some of these possible scenarios that could result in hyponastic leaf growth are depicted in Fig. 3, with either de epidermal cells directly driving differential growth (Fig. 3a), or inner (cortex) cells expanding in the basal-apical (Fig. 3b), or radial direction (Fig. 3c). Tissue-specific engineering and single cell-based omics approaches will likely help resolve cellular causalities of such organ-level responses (Han et al. 2023; Küpers et al. 2023).

## Concluding remarks

To summarize, light direction, quality, irradiance, and fluctuations over time are all perceived by plants via several different families of photoreceptors. This information is employed by the plant to respond with adequate growth adjustment. Both hypocotyls and leaves are able to bend and move upon different light signals. Growth adjustments are facilitated by local modifications in cell wall components, highly regulated by light. The same subset of genes encoding CWMPs are expressed in both hypocotyls and leaves in low R:FR. Nevertheless, hypocotyl phototropism and leaf hyponasty involve different pathways from signal perception to cell growth gradients across the tissue, being relatively cell-autonomous in phototropism but involving long-distance signaling in hyponasty. Both, however, converge on auxin-mediated regulation of cell elongation, although many of the cellular and subcellular mechanisms remain to be resolved (see Outstanding Questions).

Advances

- Intercellular air spaces facilitate the formation of a blue light gradient across the hypocotyl, enabling a phototropic response in this extremely thin tissue.
- An auxin gradient between abaxial and adaxial side is built up inside the petiole base in response to remote signaling of FR enrichment in the leaf tip and initiates upward leaf movement (hyponasty).
- Advances in plant imaging systems revealed temporal dynamics of light-driven leaf movement and elongation, thus expanding our understanding of Arabidopsis shade avoidance response kinetics beyond seedlings.
- Although different (light) signals regulate similar shade avoidance phenotypes, they partially regulate different genes and gene families to achieve this.
- Transcriptome studies have enriched our understanding of light-driven plasticity and differential growth, with especially the spatially resolved studies on dissected tissues showing how local signaling can translate into systemic or remote responses.

Outstanding questions

- What are the cellular task distributions within organs for light signaling, transduction, and growth control?
- How does light signaling through photoreceptors regulate cell wall composition and structure?
- Does differential growth rely on measurable gradients in cell wall composition and extensibility?
- How do light signaling and growth control develop over the duration of the light treatment between the differentially growing tissues?
- How conserved are regulatory mechanisms between different developmental stages?
- To what extent are currently resolved regulatory networks in Arabidopsis evolutionary conserved and translatable to crop species?

## Data Availability

No new data was generated
